# Supplementary figures and images for: Does plastic type matter? Insights into non-indigenous marine larvae recruitment under controlled conditions
Source: PeerJ. 2022 Dec 19;10:e14549. doi: 10.7717/peerj.14549 (PMC9774007; doi:10.7717/peerj.14549)

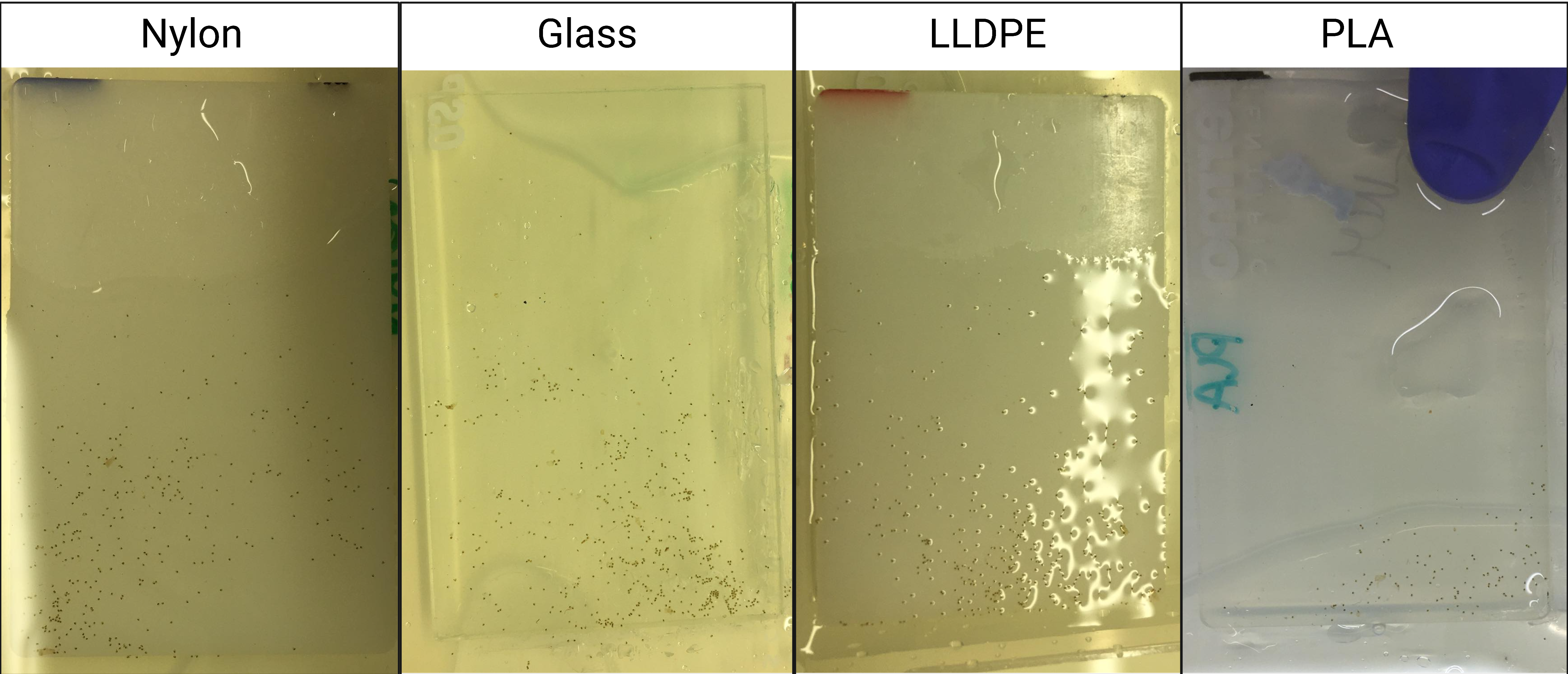

Supplement: Supplemental Information 2 — Pictures of oysters’ larvae aggregating on the different types of substrates. LLDPE: Low-Linear Density Polyethylene; PLA: Polylactic Acid. Pictures were taken with a classic camera. [file peerj-10-14549-s002.png]

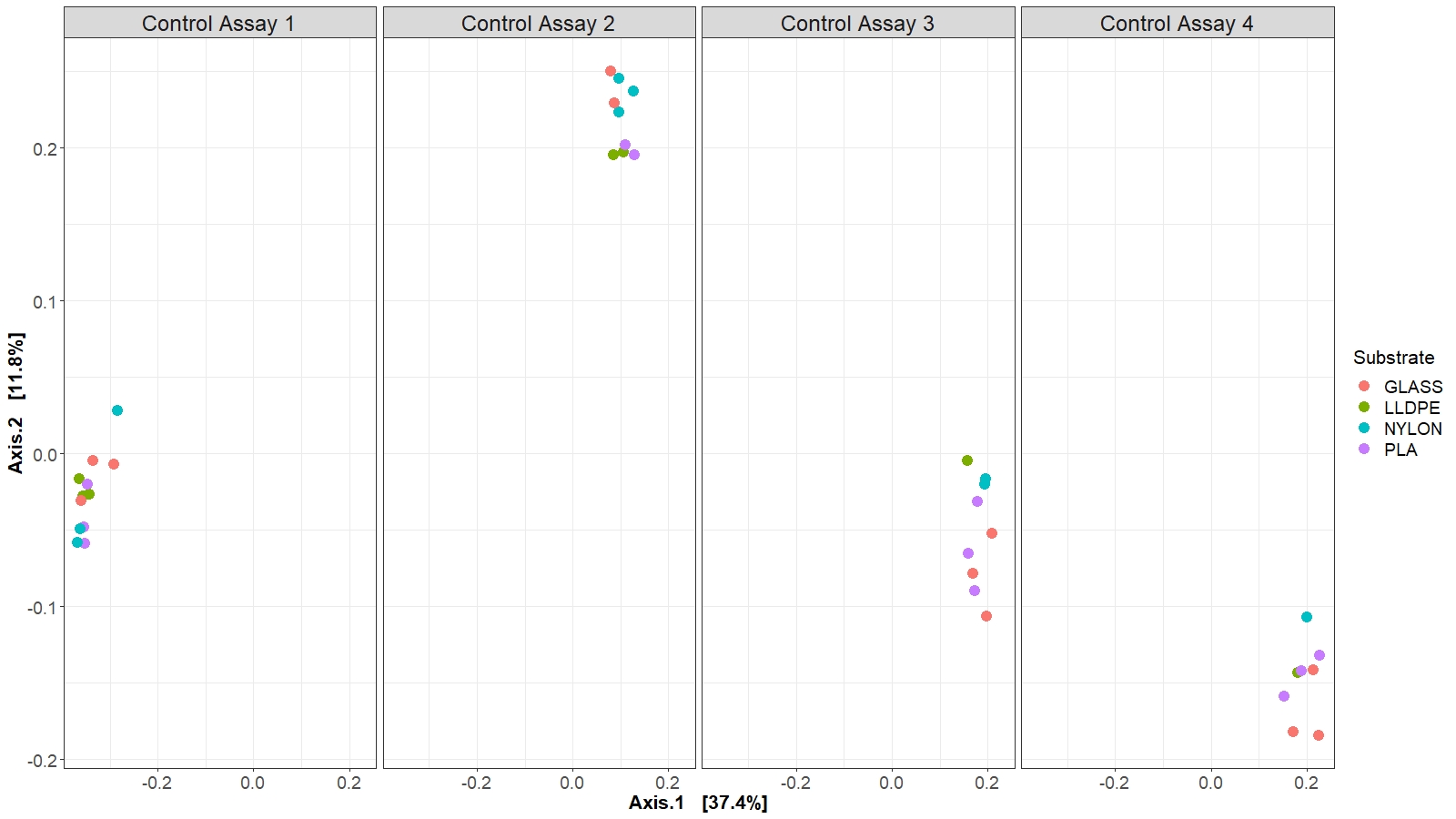

Supplement: Supplemental Information 4 — Based on weighted unifrac distance. Colors represent the various substrate types. [file peerj-10-14549-s004.jpeg]
